# Photocatalytic degradation of brilliant green and 4-nitrophenol using Ni-doped Gd(OH)_3_ nanorods

**DOI:** 10.1038/s41598-024-58688-2

**Published:** 2024-04-09

**Authors:** Shaidatul Najihah Matussin, Fazlurrahman Khan, Mohammad Hilni Harunsani, Young-Mog Kim, Mohammad Mansoob Khan

**Affiliations:** 1 Chemical Sciences, Faculty of Science, Universiti Brunei Darussalam, Jalan Tungku Link, Gadong, BE 1410, Brunei Darussalam; 2 Institute of Fisheries Science, Pukyong National University, Busan, 48513 Republic of Korea; 3 Marine Integrated Biomedical Technology Center, The National Key Research Institutes in Universities, Pukyong National University, Busan, 48513 Republic of Korea; 4 Research Center for Marine Integrated Bionics Technology, Pukyong National University, Busan, 48513 Republic of Korea; 5 Department of Food Science and Technology, Pukyong National University, Busan, 48513 Republic of Korea

**Keywords:** Gd(OH)_3_, Nickel-doped Gd(OH)_3_, 4-nitrophenol, Brilliant green, Photocatalysis, Chemistry, Materials science

## Abstract

Gadolinium hydroxide (Gd(OH)_3_) was synthesized via a microwave-assisted synthesis method. Nickel ion (Ni^2+^) was doped into Gd(OH)_3_, in which 4–12% Ni-Gd(OH)_3_ was synthesized, to study the effect of doping. The structural, optical, and morphological properties of the synthesized materials were analyzed. The crystallite sizes of the hexagonal structure of Gd(OH)_3_ and Ni-Gd(OH)_3_, which were 17–30 nm, were obtained from x-ray diffraction analysis. The vibrational modes of Gd(OH)_3_ and Ni-Gd(OH)_3_ were confirmed using Raman and Fourier-transform infrared spectroscopies. The band gap energy was greatly influenced by Ni-doping, in which a reduction of the band gap energy from 5.00 to 3.03 eV was observed. Transmission electron microscopy images showed nanorods of Gd(OH)_3_ and Ni-Gd(OH)_3_ and the particle size increased upon doping with Ni^2+^. Photocatalytic degradations of brilliant green (BG) and 4-nitrophenol (4-NP) under UV light irradiation were carried out. In both experiments, 12% Ni-Gd(OH)_3_ showed the highest photocatalytic response in degrading BG and 4-NP, which is about 92% and 69%, respectively. Therefore, this study shows that Ni-Gd(OH)_3_ has the potential to degrade organic pollutants.

## Introduction

Organic dyes have been reported to be widely found in paper, textile, and apparel industrial wastewater^1–3^. These dyes generally consist of non-biodegradable, highly poisonous, and colored pigments^4^. The dye-polluted effluents can be seen polluting aquatic environments and are harmful to living organisms^5^. Therefore, removing dyes from wastewater needs urgent attention. Furthermore, 4-nitrophenol (4-NP), among the non-colored pollutants, is known to be anthropogenic and stable in nature, which would make the mineralization or degradation of 4-NP difficult at high amounts^6^. When exposed to 4-NP, severe symptoms could develop in both human beings and animals such as vomiting, headaches, and impairment of organs^7^. Therefore, like organic dyes, removing 4-NP from the environment is crucial.

The trivalent rare-earth ions (RE^3+^) are the most common and these materials have unique properties in terms of energy storage due to their unpaired 4*f* electrons^8,9^. Moreover, RE elements have attracted attention due to their controllable structure and excellent physical and chemical properties^8–10^. These promising materials have been used in different applications for instance, these materials are used as catalysts and luminescent materials, in sensors and detectors, and biomedical devices. Moreover, RE compounds are mainly used as fluorescence labels for bio-sensing^11–15^. RE hydroxides (RE(OH)_3_) amongst others have gradually attracted attention and have been synthesized through various methods^16–20^.

Gadolinium hydroxide (Gd(OH)_3_) has shown to be a good magnetic resonance imaging (MRI) contrast agent. Recently, Gd(OH)_3_ and Gd(OH)_3_-based materials have extended their uses to computed tomography (CT) contrast agents and other applications such as photocatalysis and drug delivery^21–24^. Diverse morphologies and particle sizes of Gd(OH)_3_ and its based materials have been prepared via different synthesis methods^20,25–28^. For instance, the synthesis of Gd(OH)_3_ nanoclusters via precipitation method at 350 °C for 10 min was reported^29^. The effect of organic modifier, 3,4-dihydroxy hydrocinnamic acid (DHCA) in the synthesis was studied. Interestingly, in the absence of DHCA, the as-prepared Gd(OH)_3_ showed a rod-like structure with an average length between 30 and 40 nm. However, with the addition of DHCA, Gd(OH)_3_ showed a cluster-like structure with an average length of 700–1000 nm. On the other hand, Eu-doped Gd(OH)_3_ was also synthesized using a precipitation method^30^. Hexagonal microprism morphology with a length of 0.09–0.15 µm was obtained. The synthesis of Fe-doped Gd(OH)_3_ via hydrothermal reaction produced a rod-like morphology of Fe-doped Gd(OH)_3_^31^. Moreover, Ullah et al. synthesized Pd@Gd(OH)_3_ through hydrothermal method at 180 °C for 12 h^32^. Rod-like morphology of Pd@Gd(OH)_3_ was observed to be 100–200 nm in length and 30 nm in diameter. Au@Gd(OH)_3_ was also successfully synthesized through the hydrothermal route^25^. The synthesized Au@Gd(OH)_3_ exhibited rod-like morphology with 150 nm in length and 17 nm in diameter.

In this paper, Gd(OH)_3_ and Ni-Gd(OH)_3_ NRs were synthesized using a microwave-assisted synthesis method. To the best of the authors' knowledge, no report on the microwave-assisted synthesis of Gd(OH)_3_ and Ni-Gd(OH)_3_ has been reported. The structural, optical, and morphological properties of Gd(OH)_3_ and Ni-Gd(OH)_3_ NRs were investigated using various techniques. Furthermore, the photocatalytic degradation of 4-NP and brilliant green (BG) by using Gd(OH)_3_ and (4, 8, and 12%) Ni-Gd(OH)_3_ under UV light irradiation was studied.

## Experimental

### Chemicals used

Gadolinium nitrate hexahydrate (Gd(NO_3_)_3_·6H_2_O, 99%) and nickel nitrate hexahydrate (Ni(NO_3_)_2_·6H_2_O, 99%) were obtained from Sigma-Aldrich. Sodium hydroxide (NaOH, 99.9%) was obtained from Merck. Water was purified using aquatron (England) and used throughout the experiments. For photocatalysis experiments, brilliant green (C_27_H_34_N_2_O_4_S, 90%) and 4-nitrophenol (C_6_H_5_NO_3_, 99%) were used and obtained from Merck and Sigma-Aldrich, respectively.

### Instruments used

The synthesis of Gd(OH)_3_ and Ni-Gd(OH)_3_ was carried out using a microwave reactor (Anton Paar Monowave 400, Austria). X-ray diffractometer (XRD) with Cu Kα radiation (λ = 1.5418 Å) (Shimadzu XRD-7000), Fourier Transform-Infrared Spectroscopy (FT-IR, Shimadzu IRPrestige-21 Fourier Transform-Infrared Spectrophotometer), Raman spectrometer (NRS-5100, JASCO) and X-ray photoelectron spectroscopy (XPS, Kratos Analytical, AXIS Nova) were used to analyze the structural properties of Gd(OH)_3_ and Ni-Gd(OH)_3_. The estimation of the band gap energies of Gd(OH)_3_ and Ni-Gd(OH)_3_ was conducted using UV–Vis DRS spectroscopy (Shimadzu, UV-2600). The morphology was analyzed using field emission transmission electron microscopy (FE-TEM) and selected area electron diffraction (SAED) conducted with JEM-F200 (JEOL Ltd., Tokyo, Japan). Photocatalytic activities of 4-NP and BG degradation were carried out using a Toption (TOPT-V) photochemical reactor irradiated by a 300 W UV lamp and the absorbance of 4-NP and BG was monitored using UV–visible spectrophotometer (Shimadzu UV-1900, Japan).

### Microwave-assisted synthesis of Gd(OH)_3_ NRs

Gd(OH)_3_ NRs were synthesized using the microwave-assisted synthesis method. In brief, 15 mL of 0.05 M of Gd(NO_3_)_3_·6H_2_O aqueous solution was prepared in a microwave vessel. Next, 2.4 mL of 1 M NaOH was added dropwise into the solution. The pH of the solution was about 10. The vessel was then put in the microwave reactor in which the temperature was increased in a step-wise manner; from room temperature to 90 °C and finally to 180 °C. The temperature was maintained at 180 °C for 15 min at 850 W microwave power. Once the precipitate was formed, it was then centrifuged and washed three times with water before drying at 80 °C.

### Microwave-assisted synthesis of Ni-Gd(OH)_3_ NRs

Ni-Gd(OH)_3_ NRs were synthesized using the same method as mentioned above. A 15 mL of 0.05 M aqueous Gd(NO_3_)_3_·6H_2_O solution was prepared and a specific amount of Ni(NO_3_)_2_·6H_2_O was added to prepare 4, 8, and 12% Ni-Gd(OH)_3_ NRs. NaOH with a concentration of 1 M was then added dropwise into the solution and the pH of the solution was about 10. Subsequently, the synthesis reaction was heated in a step-wise manner to 90 °C and finally to 180 °C and maintained at 180 °C for 15 min at 850 W microwave power. The precipitate was then centrifuged and washed three times with distilled water before it was dried at 80 °C. The products were coded as 4% Ni-Gd(OH)_3_, 8% Ni-Gd(OH)_3_, and 12% Ni-Gd(OH)_3_.

### Photocatalytic degradation of brilliant green and 4-nitrophenol

Photocatalytic degradation of BG dye and 4-NP using Gd(OH)_3_ and 4, 8, and 12% Ni-Gd(OH)_3_ NRs under UV light irradiation were investigated. In brief, 10 mg of Gd(OH)_3_ and 4, 8, and 12% Ni-Gd(OH)_3_ NRs were mixed with 50 mL of the respective pollutants: 10 ppm of BG dye or 4-NP solutions. The sample mixture was sonicated for 3 min and stirred in the dark for another 3 min. Then, the reaction solutions were continuously stirred and irradiated with UV light (300 W) for 5 h. The absorbance of the BG or 4-NP solution at λ_max_ of 620 and 316 nm, respectively, was taken every 1 h to observe the photocatalysis progress for a total of five hours. The percentage of photocatalytic BG dye or 4-NP degradation was obtained using the following equation (Eq. 1):

1 $$\% photocatalytic \;BG \;dye \;or \;4-NP \;degradation=\frac{({A}_{blank}-{A}_{sample})}{{A}_{blank}}\times 100$$

where A_blank_ is the absorbance of BG or 4-NP only and A_sample_ is the absorbance of BG or 4-NP after photocatalytic degradation reaction with the respective catalyst.

## Results and discussion

### X-ray diffraction and Fourier Transform infrared spectroscopy

**Figure 1:**
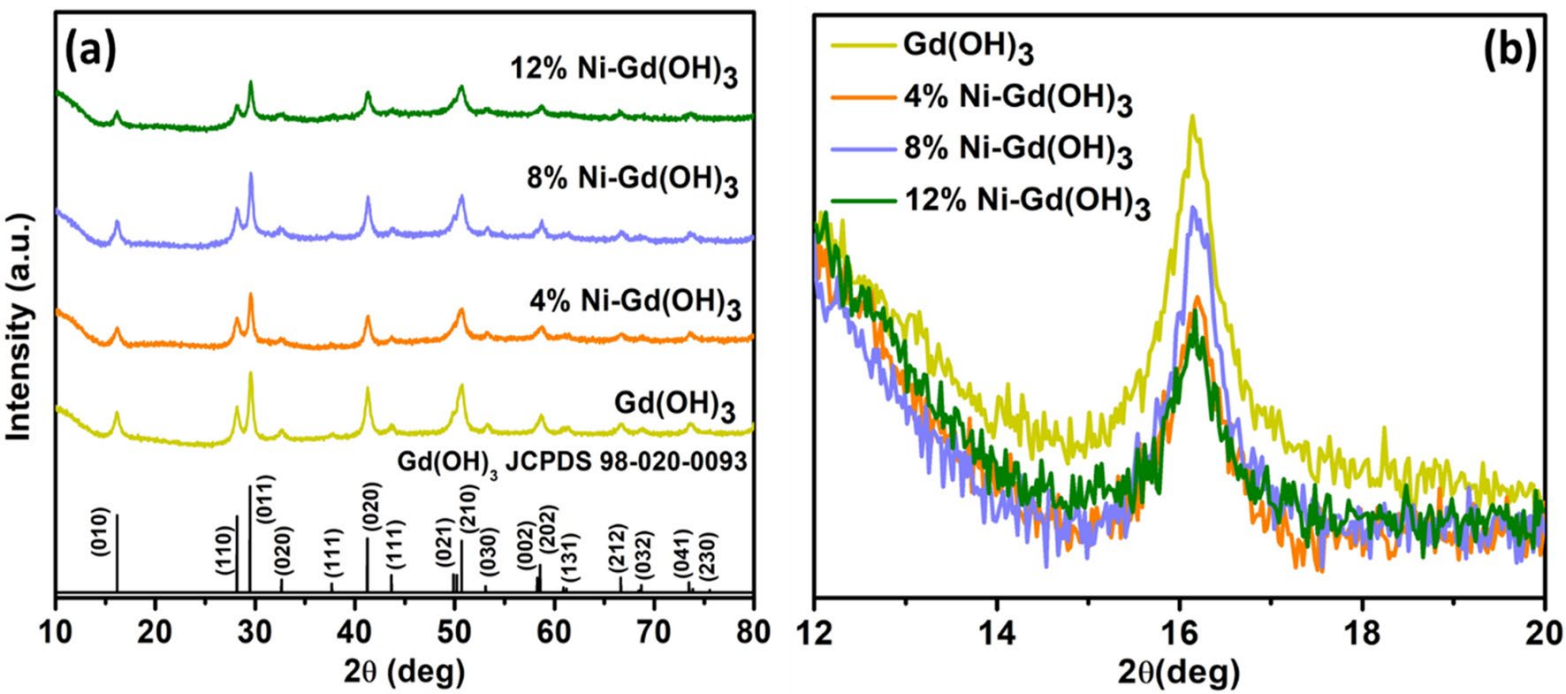
(**a**) XRD pattern and (**b**) zoom-in view of the (010) plane of Gd(OH)_3_ and 4, 8, and 12% Ni-Gd(OH)_3_ NRs.

XRD analysis of Gd(OH)_3_ and Ni-Gd(OH)_3_ NRs was conducted and the XRD patterns are presented in Fig. 1a. Pure Gd(OH)_3_ showed a hexagonal phase with a space group of P6_3_/m and the peaks at 2θ = 16.22, 28.07, 29.53, 32.75, and 37.68° are associated with (010), (110), (011), (020), and (111) planes, respectively (JCPDS 98-020-0093)^33^. No additional peaks were observed after doping with Ni^2+^ ions, suggesting the successful incorporation of Ni^2+^ into the Gd(OH)_3_ lattice. Figure 1b shows the zoom-in view of the (010) plane of Gd(OH)_3_ and Ni-Gd(OH)_3_. No significant shift was observed with Ni-doping which suggests that the Ni-doping did not change the Gd(OH)_3_ lattice. However, the peak intensity was seen to decrease. This might be due to the reduction in the crystallinity of Gd(OH)_3_^34^.

The effect of Ni doping on the structural properties of Gd(OH)_3_ was studied by the estimation of the average crystallite sizes of Gd(OH)_3_ and Ni-Gd(OH)_3_ using the Debye–Scherrer’s equation (Eq. 2) and their average lattice strains were also calculated (Eq. 3)^7,35^:

2 $${\text{D}} = {\text{k}}\lambda /\beta {\text{ cos }}\theta$$

3 $$\varepsilon =\frac{{\beta }_{hkl}}{4tan\theta }$$

where *λ* represents the wavelength of the x-ray, θ indicates Bragg’s angle, and β is the full width at half maximum of the characteristic peaks. The calculated average crystallite size of Gd(OH)_3_ was found to be 30.08 nm (Table 1). When 4% Ni was doped into Gd(OH)_3_, the average crystallite size was reduced to 20.84 nm. Further doping showed similar crystallite size (21.59 nm) for 8% Ni-Gd(OH)_3_. However, 12% Ni-Gd(OH)_3_ showed smaller crystallite size of 17.34 nm. However, the lattice parameters and cell volumes of Gd(OH)_3_ and Ni-Gd(OH)_3_ show insignificant change. Nonetheless, the average lattice strain increased with Ni-doping from 0.0012 to 0.0021. The Ni-doping has mainly influenced the average crystallite size and the lattice strain. However, the Ni-doping was observed to maintain the Gd(OH)_3_ lattice without significantly affecting the lattice parameter and the cell volume.

**Table 1:** Average crystallite size (nm), lattice parameter (Å), cell volume (Å^3^), and lattice strain(ε) of Gd(OH)_3_ and Ni-Gd(OH)_3_ NRs.

Samples	Average crystallite size (nm)	Lattice parameter (Å)		Cell volume (Å^3^)	Average lattice strain (ε)
		*a*	*c*		
Gd(OH)_3_	30.08	6.34	4.18	145.51	0.0012
4% Ni-Gd(OH)_3_	20.84	6.32	4.18	144.59	0.0017
8% Ni-Gd(OH)_3_	21.59	6.31	4.18	144.13	0.0019
12% Ni-Gd(OH)_3_	17.34	6.31	4.18	144.13	0.0021

### Fourier transform infrared spectroscopy and Raman spectroscopy

**Figure 2:**
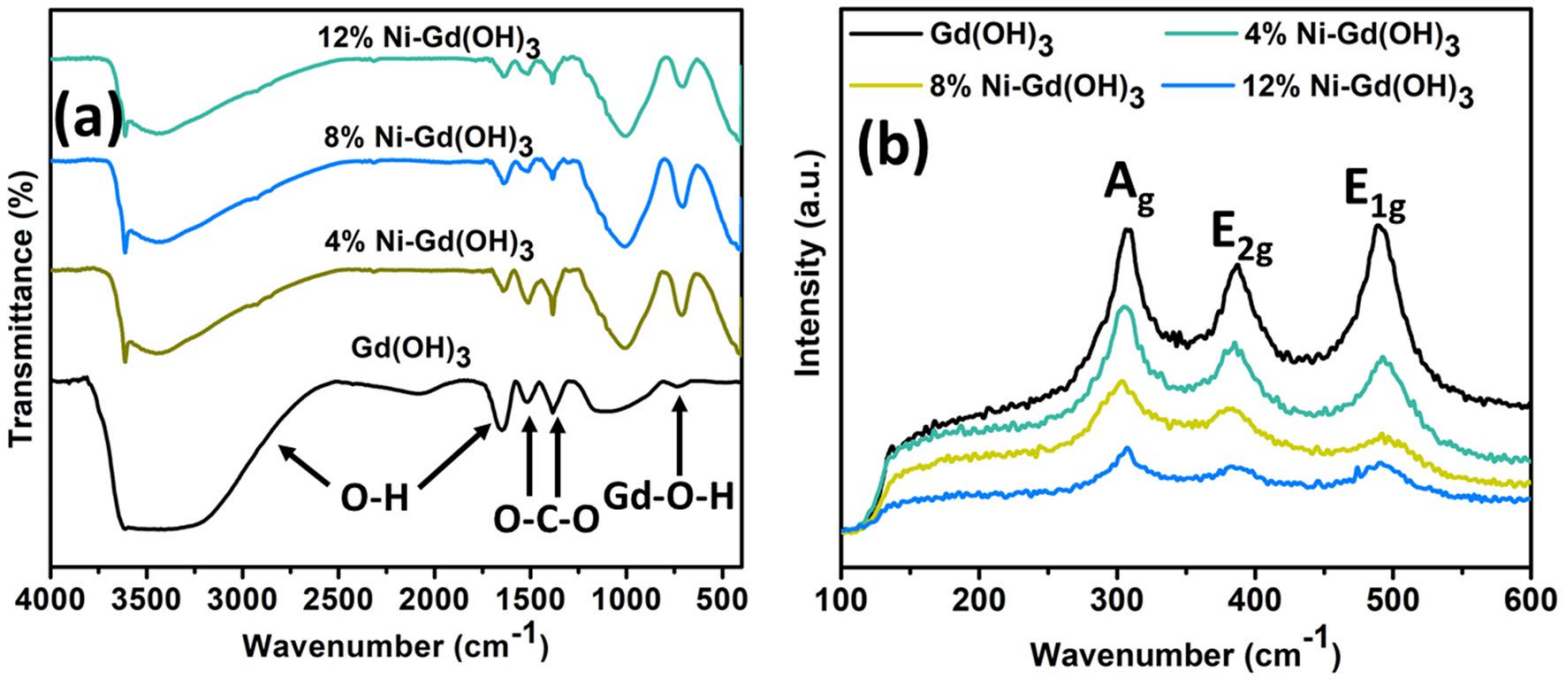
(**a**) FT-IR and (**b**) Raman spectra of Gd(OH)_3_ and 4, 8, and 12% Ni-Gd(OH)_3_ NRs.

Figure 2(a) shows the FT-IR spectra of Gd(OH)_3_ and Ni-Gd(OH)_3_ NRs. All samples showed a bending vibration of Gd–O–H in the range of 600–750 cm^-1^, which confirmed the synthesis of Gd(OH)_3_ and Ni-Gd(OH)_3_. The common symmetric and asymmetric stretching of O–C–O in Gd(OH)_3_ can be observed in the range of 1370–1530 cm^-1^^36^. The O–H vibration band and the stretching and bending of O–H vibration were observed at ~ 1600 cm^-1^ and ~ 3500 cm^-1^, respectively^37^. Raman spectra of Gd(OH)_3_, 4%, 8%, and 12% Ni-Gd(OH)_3_ are shown in Fig. 2b. Pure Gd(OH)_3_ showed three main Raman peaks assigned to A_g_ translatory, E_2g_ translatory, and E_1g_ liberation modes which are located at 308.85, 387.34, and 490.52 cm^-1^, respectively^37^. One should note that, 4A_g_, 2E_1g_, and 5E_2g_ are known to be Raman active for hexagonal phase Gd(OH)_3_ with P6_3_/m space group^37^. The Raman peak intensity was decreased when 4% Ni was doped into Gd(OH)_3_. Expectedly, when more Ni-doping was incorporated the Raman peak intensity decreased further. This suggests that with more Ni-doping, the distortion of the lattice periodicity and long-range translational crystal symmetry caused by the induced defects occurred in the crystal lattice^38^.

### Transmission electron microscopy

**Figure 3:**
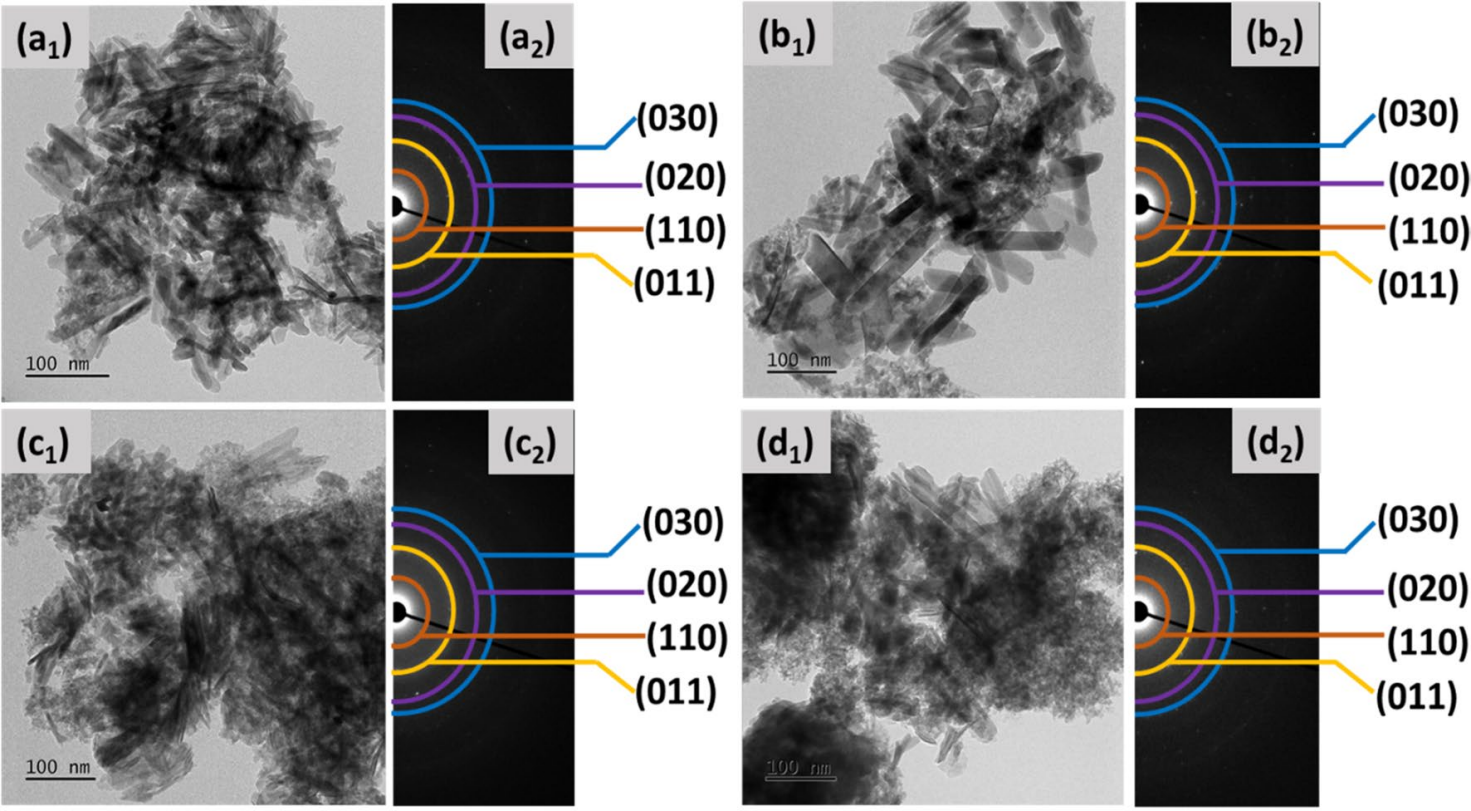
TEM images of (**a**_**1**_) Gd(OH)_3_, (**b**_**1**_) 4% Ni-Gd(OH)_3_, (**c**_**1**_) 8% Ni-Gd(OH)_3_, and (**d**_**1**_) 12% Ni-Gd(OH)_3_ and SAED patterns of **(a**_**2**_**)** Gd(OH)_3_, **(b**_**2**_**)** 4% Ni-Gd(OH)_3_, **(c**_**2**_**)** 8% Ni-Gd(OH)_3_, and (**d**_**2**_) 12% Ni-Gd(OH)_3_.

TEM images of Gd(OH)_3_, 4%, 8%, and 12% Ni-Gd(OH)_3_ are shown in Fig. 3a_1_–d_1_, and all synthesized materials displayed rod-like morphology. This indicates that the addition of Ni-doping has no major influence on the morphology. However, the particle size was influenced by the Ni doping. Gd(OH)_3_ showed an average length of 38 nm and a diameter of 12 nm (Table 2). When 4% Ni was incorporated, the particle length was increased to 76 nm and the diameter was increased to 26 nm. This suggests that Ni-doping has some influence on the particle size of Gd(OH)_3_ as reported by Kumar et al.^39^ However, further increase in Ni doping has no more influence on the particle lengths. The average particle lengths for 8% Ni-Gd(OH)_3_ and 12% Ni-Gd(OH)_3_ were 76 nm and 75 nm and the average diameters were 13 and 14 nm, respectively. This might be due to the hindering of crystal growth at a certain level of doping, in which, in this case, 12% Ni might hinder the crystal growth^40^.

**Table 2:** Average particle sizes from TEM and the band gap energy of Gd(OH)_3_, and Ni-Gd(OH)_3_ NRs (*L = length and D = diameter).

Sample	Average particle size (nm) from TEM	Band gap energy (eV)
Gd(OH)_3_	38 (L), 12 (D)	5.00
4% Ni-Gd (OH)_3_	76 (L), 26 (D)	4.37
8% Ni-Gd (OH)_3_	76 (L), 13 (D)	3.75
12% Ni-Gd (OH)_3_	75 (L), 14 (D)	3.03

The phase and diffraction patterns of Gd(OH)_3_, 4%, 8%, and 12% Ni-Gd(OH)_3_ NRs were examined by SAED analysis as shown in Figs. 3a_2_–d_2_. It was observed that the synthesized materials exhibit four broad rings, which are attributed to the (110), (011), (020), and (030) reflections of the hexagonal Gd(OH)_3_ (P6_3_/m) structure. This is in agreement with the XRD results (Section “X-ray diffraction and Fourier Transform Infrared spectroscopy”).

### UV–vis diffuse reflectance spectroscopy

**Figure 4:**
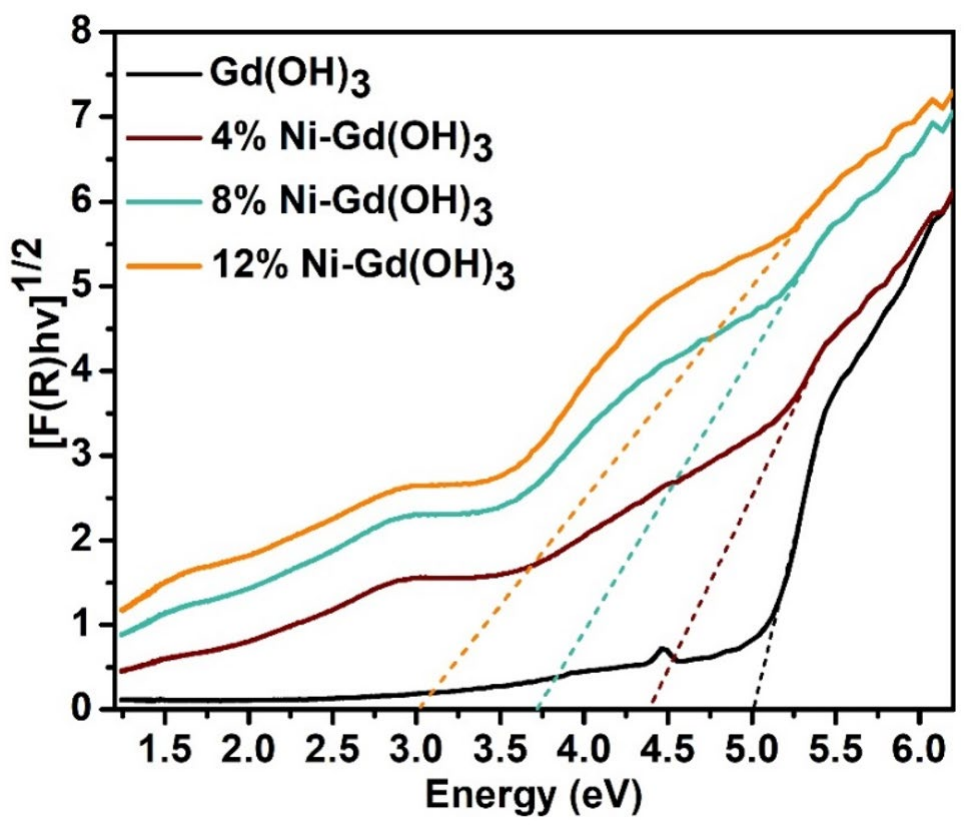
Tauc plot obtained from the Kubelka–Munk function for band gap energy estimation of Gd(OH)_3_ and 4, 8, and 12% Ni-Gd(OH)_3_ NRs.

The band gap energy of Gd(OH)_3_ and Ni-Gd(OH)_3_ was estimated from the Tauc plot (Fig. 4) that was obtained from the Kubelka–Munk equation (Eq. 4).

4 $$F\left(R\right)={\left(\frac{{(1-R)}^{2}}{2R}\times h\nu \right)}^\frac{1}{2}$$

where *R* is the measured absolute reflectance of the samples. The band gap can be obtained from the plots of [F(*R*)*hv*]^1/2^ versus *hv*.

The band gap energy of pure Gd(OH)_3_ is 5.00 eV which is in good agreement with literature^41^. Interestingly, the band gap energy decreased to 4.37 eV in the case of 4% Ni-Gd(OH)_3_. This suggests that the Ni-doping has affected the optical band gap energy of Gd(OH)_3_. Further increase in the percentage of Ni-doping has led to a decrease in the band gap energy even further. Therefore, 8% Ni-Gd(OH)_3_ and 12% Ni-Gd(OH)_3_ showed band gap energies of 3.75 and 3.03 eV. The band gap energies of all samples were tabulated in Table 2.

### X-ray photoelectron spectroscopy

**Figure 5:**
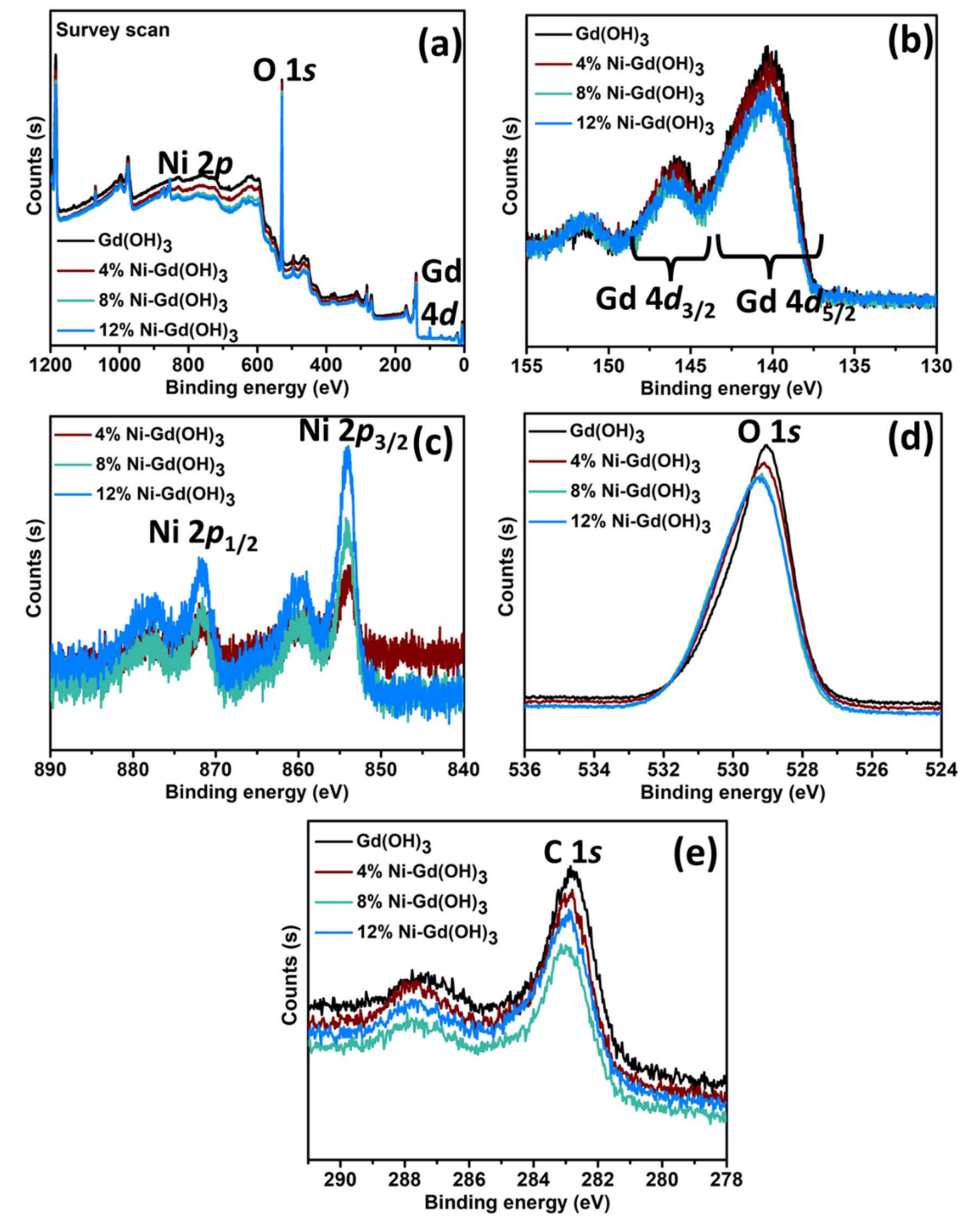
XPS spectra of Gd(OH)_3_ and Ni-Gd(OH)_3_ NRs: (**a**) Survey scan, (**b**) Gd 4*d*, **(c)** Ni 2*p*, (**d**) O 1*s*, and (**e**) C 1*s*.

The chemical state and the electronic structure of the elements in Gd(OH)_3_ and Ni-Gd(OH)_3_ were analyzed using XPS. Figure 5a shows the survey scan spectra of the synthesized materials confirming the presence of Gd 4*d*, Ni 2*p*, and O 1*s*. The Gd 4*d* core level peak is shown in Fig. 5b. Two major peaks at approximately 140.3 and 146.1 eV were observed, corresponding to Gd^3+^ 4*d*_3/2_ and Gd^3+^ 4*d*_5/2_, respectively^32^. No peak shift was observed for all the synthesized materials. Figure 5c shows the XPS spectra of two prominent Ni 2*p* peaks of 4% Ni-Gd(OH)_3_, 8% Ni-Gd(OH)_3_ and 12% Ni-Gd(OH)_3_ NRs. The two prominent peaks at 853.9 and 871.6 eV correspond to the Ni 2*p*_3/2_ and Ni 2*p*_1/2_, respectively^42^. Furthermore, the peak intensity was increased with more Ni-doping. The peak positions of 878.7 and 860.4 eV represent Ni 2*p*_3/2_ and Ni 2*p*_1/2_ satellite peaks^43^.

The XPS spectrum of O 1*s* can be seen in Fig. 5d, in which all the samples exhibit one major peak. The peak at approximately 529.0 eV indicates the OH^-^ anion^44^. Slight shifts and changes in the peak position and intensity were observed for Ni-Gd(OH)_3_. The typical C 1*s* peaks at 282.9 eV were observed in the spectra (Fig. 5e). XPS intensities were observed to vary with the incorporation of Ni^2+^ ions. The atomic concentrations of C 1*s*, O 1*s*, Gd 4*d*, and Ni 2*p* are listed in Table 3.

**Table 3:** The atomic concentration of C 1*s*, O 1*s*, Gd 4*d*, and Ni 2*p* of Gd(OH)_3_ and Ni-Gd(OH)_3_ NRs.

Samples	Atomic concentrations (%)			
	C 1*s*	O 1*s*	Gd 4*d*	Ni 2*p*
Gd (OH)_3_	7.6	86.5	5.0	–
4% Ni-Gd (OH)_3_	6.7	85.2	4.7	2.9
8% Ni-Gd (OH)_3_	5.7	84.7	4.4	4.7
12% Ni-Gd (OH)_3_	6.4	82.9	4.2	6.0

## Applications

### Photocatalytic degradation of BG dye

**Figure 6:**
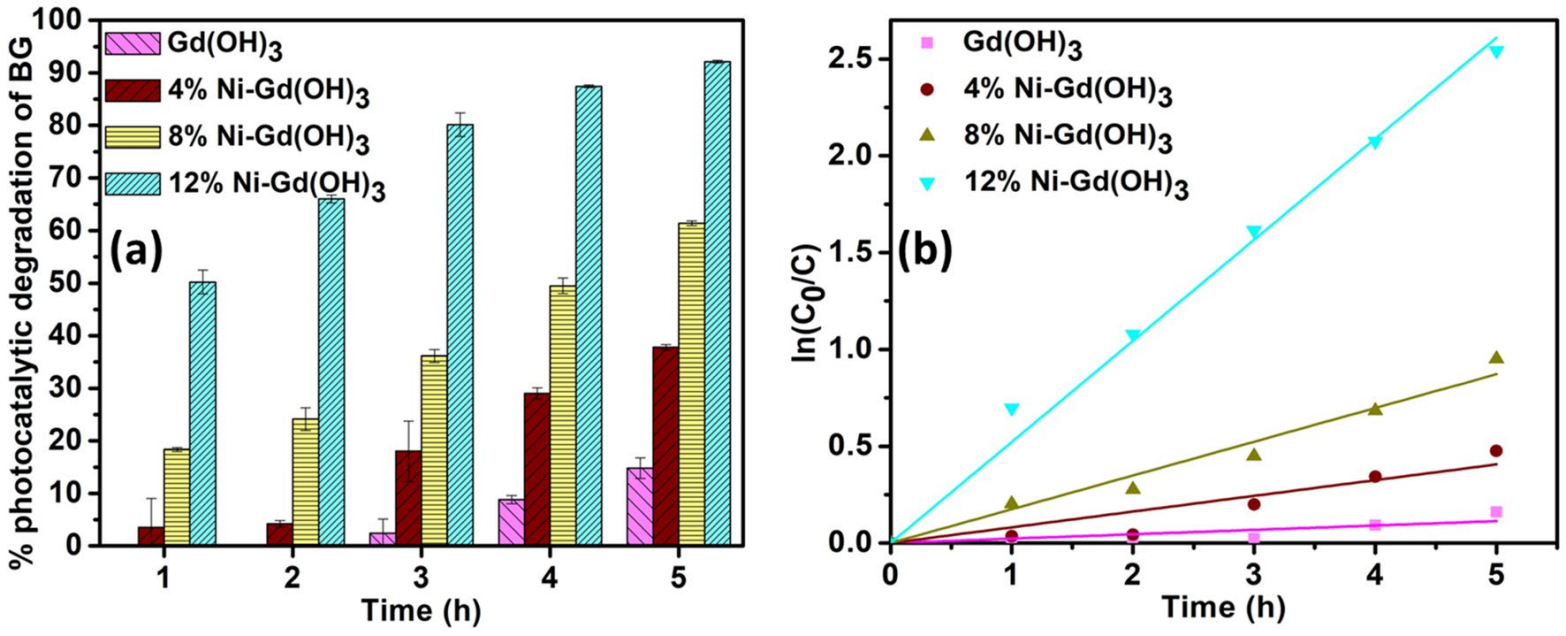
(**a**) Average percentage and (**b**) ln(C_0_/C) plot of photocatalytic degradation of BG using Gd(OH)_3_ and Ni-Gd(OH)_3_ NRs under UV light irradiation.

The photocatalytic degradation of BG using Gd(OH)_3_ and Ni-Gd(OH)_3_ was studied under the irradiation of UV light for 5 h (Fig. 6). The progress of the photocatalytic activity was monitored every hour by measuring the absorbance of the treated BG solution at λ_max_ = 620 nm. The experiment was conducted in triplicates and the average percentage of the photocatalysis is shown in Fig. 6a.

At the 1st and 2nd hour, Gd(OH)_3_ showed no photocatalytic response but there was a slight response at the 3rd and 4th hour. It was finally able to degrade about 14.81 ± 1.96% at the 5th hour of UV light irradiation. 4% Ni-Gd(OH)_3_ showed a slightly better photocatalytic response even though it was only able to degrade less than 30% from 1st to 4th hour. It finally degraded about 37.82 ± 0.51% of BG at the 5th hour. The photocatalytic response was seen increasing with more Ni-doping, as can be observed from 8% Ni-Gd(OH)_3_. At the 1st hour, 8% Ni-Gd(OH)_3_ degraded about 18.35 ± 0.39% and showed about 61.39 ± 0.47% degradation at the 5th hour. Moreover, the percentage photocatalytic degradation of BG was observed to be higher for 12% Ni-Gd(OH)_3_, which showed about 50.20 ± 2.28% at the 1st hour and 92.14 ± 0.29% at the 5th hour. This suggests that, when more Ni^2+^ was doped, the photocatalytic degradation of BG increased. Based on their band gap energies, more Ni doping resulted in the reduction of the band gap energy (i.e., 5.00–3.30 eV). The doping might help in creating defect states in the band gap which enables UV light absorption and thus inhibits rapid charge carrier recombination^45^. Table 4 shows the average percentage of the photocatalytic degradation of BG using Gd(OH)_3_ and 4, 8, and 12% Ni-Gd(OH)_3_ NRs.

**Table 4:** The average percentage of photocatalytic activities of Gd(OH)_3_ and 4, 8, and 12% Ni-Gd(OH)_3_ NRs for BG degradation under UV light irradiation.

	% photocatalytic degradation of BG				
	1 h	2 h	3 h	4 h	5 h
Gd(OH)_3_	–	–	2.40 ± 1.71	8.82 ± 0.79	14.81 ± 1.96
4% Ni-Gd (OH)_3_	3.53 ± 2.51	4.23 ± 0.61	18.04 ± 5.73	29.02 ± 1.07	37.82 ± 0.51
8% Ni-Gd (OH)_3_	18.35 ± 0.39	24.13 ± 2.16	36.16 ± 1.23	49.48 ± 1.49	61.39 ± 0.47
12% Ni-Gd (OH)_3_	50.20 ± 2.28	66.01 ± 0.77	80.11 ± 2.24	87.43 ± 0.24	92.14 ± 0.29

Moreover, the photocatalytic activity of Gd(OH)_3_ and Ni-Gd(OH)_3_ NRs against BG was studied by applying the pseudo-first-order reactions (Eq. 5):

5 $$ln\frac{{C}_{0}}{C}=Kt$$

The rate constant of the first order (*K*) is expressed in 1/h. Figure 6b presents the pseudo-first-order reactions of the photocatalytic degradation of BG activity of Gd(OH)_3_ and Ni-Gd(OH)_3_ NRs. The rate constants of Gd(OH)_3_, 4% Ni-Gd(OH)_3_, 8% Ni-Gd(OH)_3_, and 12% Ni-Gd(OH)_3_ NRs were estimated to be 0.0226, 0.0812, 0.1744, and 0.5220 h^-1^, respectively. It was observed that 12% Ni-Gd(OH)_3_ NRs showed the highest reaction rate constant.

### Photocatalytic degradation of 4-nitrophenol

**Figure 7:**
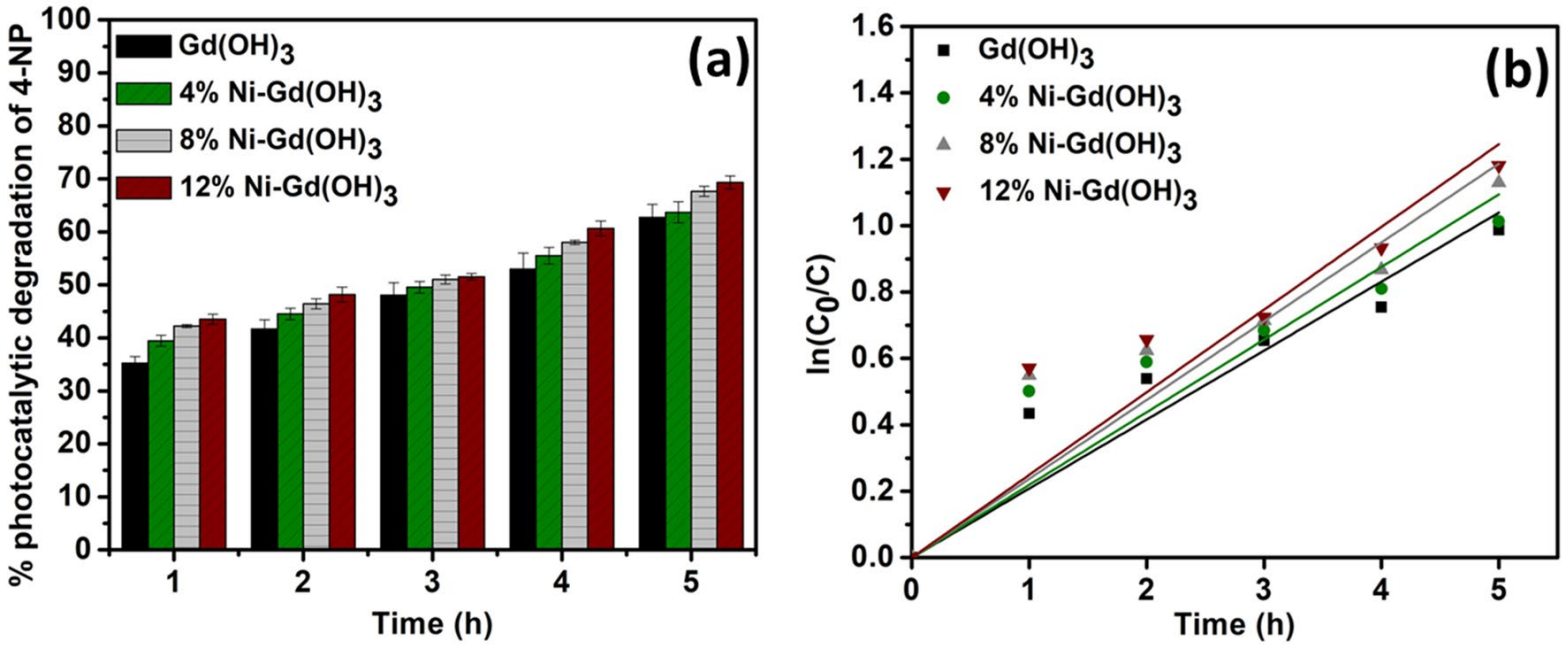
(**a**) Average percentage and (**b**) ln(C_0_/C) plot of photocatalytic degradation of 4-NP using Gd(OH)_3_ and Ni-Gd(OH)_3_ NRs under UV light irradiation.

Photocatalytic degradation of 4-NP was also investigated using Gd(OH)_3_ and Ni-Gd(OH)_3_ NRs under the irradiation of UV light (Fig. 7a). Similarly, at every hour, the absorbance of the treated 4-NP solution was taken and measured to observe the photocatalytic response. The readings were taken three times to ensure repeatability.

Under the UV light irradiation, the photocatalytic response of Gd(OH)_3_ throughout the experiment was expected. It showed the lowest response amongst the samples which was about 62.74 ± 2.44% of the photocatalytic degradation of 4-NP. This might be due to its wide band gap (5.00 eV). When 4% Ni was doped, the photocatalytic response was increased slightly (63.69 ± 1.99%), suggesting the effect of Ni^2+^ doping. Moreover, the reduction in the band gap energy from 5.00 eV to 4.37 eV might cause a slight enhancement in the separation of the photogenerated electrons (e^-^) and holes (h^+^). Further increase in the percentage of Ni^2+^ doping has shown better photocatalytic degradation of 4-NP. In the case of 8% Ni-Gd(OH)_3_, the overall photocatalytic response was about 67.67 ± 0.95% whereas 12% Ni-Gd(OH)_3_ showed about 69.32 ± 1.25% of 4-NP degradation (Table 5). The band gap energies of both materials decreased with more Ni^2+^ doping, which were about 3.75 and 3.03 eV, respectively. Therefore, the efficient photocatalytic response might be due to their lower band gap energies.

**Table 5:** The average percentage of photocatalytic activities of Gd(OH)_3_ and 4, 8, and 12% Ni-Gd(OH)_3_ NRs for 4-NP degradation under UV light irradiation.

	% Photocatalytic degradation of 4-NP				
	1 h	2 h	3 h	4 h	5 h
Gd (OH)_3_	35.22 ± 1.25	41.67 ± 1.73	48.03 ± 2.39	52.98 ± 3.03	62.74 ± 2.44
4% Ni-Gd (OH)_3_	39.45 ± 1.02	44.52 ± 1.09	49.55 ± 1.08	55.52 ± 1.57	63.69 ± 1.99
8% Ni-Gd (OH)_3_	42.24 ± 0.29	46.41 ± 0.96	51.03 ± 0.82	58.00 ± 0.39	67.67 ± 0.95
12% Ni-Gd (OH)_3_	43.52 ± 0.95	48.16 ± 1.42	51.52 ± 0.62	60.66 ± 1.41	69.32 ± 1.25

The kinetic study was conducted based on the photocatalytic activity of Gd(OH)_3_ and Ni-Gd(OH)_3_ NRs against 4-NP (Eq. 5). Figure 7b presents the pseudo-first-order reactions of the photocatalytic degradation of BG activity of Gd(OH)_3_ and Ni-Gd(OH)_3_ NRs. The rate constants of Gd(OH)_3_, 4% Ni-Gd(OH)_3_, 8% Ni-Gd(OH)_3_, and 12% Ni-Gd(OH)_3_ NRs were estimated to be 0.2078, 0.2189, 0.2374, and 0.2490 h^-1^, respectively. It was observed that, with Ni-doping, the reaction rates were increased, and 12% Ni-Gd(OH)_3_ NRs showed the highest reaction rate constant.

Therefore, based on both photocatalysis experiments, 12% Ni-Gd(OH)_3_ showed the highest photocatalytic degradation of BG and 4-NP. The difference in the effectiveness of 12% Ni-Gd(OH)_3_ in both cases might be due to the nature of the two compounds. Moreover, the narrow band gap energy of 12% Ni-Gd(OH)_3_ (3.03 eV) might absorb UV light more efficiently and can photogenerate e^-^/h^+^ pairs effectively. One study reported on the catalytic photodegradation of Congo red using lanthanide hydroxides (Ln = Nd, Sm, Eu, Gd, Tb, and Dy)^46^. The removal efficiencies of Ln(OH)_3_ are more than 90% after 1800 min. However, this current study shows improved photocatalytic efficiency where the photocatalytic degradation of BG using Ni-Gd(OH)_3_ showed a degradation of BG over 90% in 5 h. This might be also due to the recombination of the photogenerated e^-^/h^+^ pairs being hindered. The photogenerated e^-^/h^+^ pairs would react with adsorb O_2_ and H_2_O to form O_2_^•-^ and OH^•^ radicals, respectively. In general, these radicals are responsible for the degradation of pollutants. However, based on the literature, O_2_^•-^ radicals are mainly responsible for the degradation of dyes, whereas OH^•^ radicals are responsible for the degradation of 4-NP^47–49^. Therefore, the difference in the response of 12% Ni-Gd(OH)_3_ in the degradation of BG and 4-NP might be due to these radicals. The role of O_2_^•-^ radicals might be more prominent during the photocatalytic degradation of BG and in the case of the photocatalytic degradation of 4-NP, OH^•^ radicals might be the more reactive species.

## Conclusions

Gd(OH)_3_ and Ni-Gd(OH)_3_ NRs were successfully synthesized using the microwave-assisted synthesis method. The properties of the synthesized materials such as the structural, optical, and morphological properties, were analyzed using different instruments. The hexagonal structure of Gd(OH)_3_ and Ni-Gd(OH)_3_ with average crystallite sizes between 17 and 30 nm was obtained from XRD. The presence of the vibrational bands of Gd(OH)_3_ and Ni-Gd(OH)_3_ was confirmed by Raman and FT-IR spectroscopies. The band gap energy of Gd(OH)_3_ and Ni-Gd(OH)_3_ were reduced with Ni-doping in which the values decreased from 5.00 to 3.03 eV. TEM images showed nanorod-shaped Gd(OH)_3_ and Ni-Gd(OH)_3_ with increased particle size when doping with Ni^2+^. Photocatalytic degradations of 4-NP and BG under UV light irradiation were carried out and 12% Ni-Gd(OH)_3_ showed the highest photocatalytic response which is about 92% and 69%, respectively. Therefore, Ni-Gd(OH)_3_ is a promising material for the degradation of organic pollutants.

## Data Availability

All data generated or analyzed during this study are included in the manuscript.
